# Karyotype alteration generates the neoplastic phenotypes of SV40-infected human and rodent cells

**DOI:** 10.1186/s13039-015-0183-y

**Published:** 2015-10-22

**Authors:** Mathew Bloomfield, Peter Duesberg

**Affiliations:** Department of Molecular and Cell Biology, Donner Laboratory, University of California at Berkeley, Berkeley, CA USA

**Keywords:** Cancer-specific reproductive autonomy, Immortality, Preneoplastic aneuploidy, Individuality of cancer phenotypes and transcriptomes, Clonal karyotypes of cancers, Speciation theory of carcinogenesis

## Abstract

**Background:**

Despite over 50 years of research, it remains unclear how the DNA tumor viruses SV40 and Polyoma cause cancers. Prevailing theories hold that virus-coded Tumor (T)-antigens cause cancer by inactivating cellular tumor suppressor genes. But these theories don’t explain four characteristics of viral carcinogenesis: (1) less than one in 10,000 infected cells become cancer cells, (2) cancers have complex individual phenotypes and transcriptomes, (3) recurrent tumors without viral DNA and proteins, (4) preneoplastic aneuploidies and immortal neoplastic clones with individual karyotypes.

**Results:**

As an alternative theory we propose that viral carcinogenesis is a form of speciation, initiated by virus-induced aneuploidy. Since aneuploidy destabilizes the karyotype by unbalancing thousands of genes it catalyzes chain reactions of karyotypic and transcriptomic evolutions. Eventually rare karyotypes evolve that encode cancer-specific autonomy of growth. The low probability of forming new autonomous cancer-species by random karyotypic and transcriptomic variations predicts individual and clonal cancers. Although cancer karyotypes are congenitally aneuploid and thus variable, they are stabilized or immortalized by selections for variants with cancer-specific autonomy. Owing to these inherent variations cancer karyotypes are heterogeneous within clonal margins. To test this theory we analyzed karyotypes and phenotypes of SV40-infected human, rat and mouse cells developing into neoplastic clones. In all three systems we found (1) preneoplastic aneuploidies, (2) neoplastic clones with individual clonal but flexible karyotypes and phenotypes, which arose from less than one in 10,000 infected cells, survived over 200 generations, but were either T-antigen positive or negative, (3) spontaneous and drug-induced variations of neoplastic phenotypes correlating 1-to-1 with karyotypic variations.

**Conclusions:**

Since all 14 virus-induced neoplastic clones tested contained individual clonal karyotypes and phenotypes, we conclude that these karyotypes have generated and since maintained these neoplastic clones. Thus SV40 causes cancer indirectly, like carcinogens, by inducing aneuploidy from which new cancer-specific karyotypes evolve automatically at low rates. This theory explains the (1) low probability of carcinogenesis per virus-infected cell, (2) the individuality and clonal flexibility of cancer karyotypes, (3) recurrence of neoplasias without viral T-antigens, and (4) the individual clonal karyotypes, transcriptomes and immortality of virus-induced neoplasias - all unexplained by current viral theories.

## Background

The DNA tumor viruses SV40 and Polyoma are very efficient carcinogens in immune-tolerant animals and in cultured animal and human cells [1–3]. But, despite over 50 years of research the mechanism of viral carcinogenesis is still unclear. The currently prevailing theories hold that non-structural viral proteins, termed Tumor (T)-antigens, cause cancer by inactivating cellular tumor suppressor genes [4–7]. However, these viral theories do not explain the following four characteristics of viral carcinogenesis.

1. *Less than 1 in 10,000 virus-infected cells form an immortal neoplastic clone.* Even under optimal experimental conditions SV40 transforms only one in over 10,000 infected human (if any [8]) or animal cells into an immortal neoplastic clone [2, 3, 8–25] (see also Results). According to their clonal origins new neoplastic clones only manifest in infected cultures after delays of several weeks to months following infection [9, 10, 12, 13, 15, 16, 26–28] (see also Results). Likewise tumors develop in animals only 3 to 24 months after injection of viruses [1, 29–31], or after transfection with cloned viral DNAs [32], or after the birth of animals with transgenic viral genes [33–35]. The low probability and late appearance of immortal neoplastic clones indicate that viral genes are not sufficient for neoplastic transformation and immortalization. But the rare clonogenic event that generates and immortalizes clonal cancers from mortal somatic cells is still unknown.
2. *Virus-induced tumors and neoplastic clones have individual rather than virus-coded phenotypes and transcriptomes.* Paradoxically, in view of the virus-cancer theory, viral tumors [1, 30, 36–38] and neoplastic clones formed in vitro [3, 9, 10, 12, 13, 15, 16, 27, 28, 39–44] have complex individual clonal phenotypes and transcriptomes, rather than common virus-specific phenotypes. The individuality of Polyoma- and SV40 virus-induced tumors even from the same tissue of origin is in fact the reason why the two viruses were surnamed ‘Polyoma viruses’ - many (= poly) different types of carcin*omas* [1, 3, 45, 46]. Accordingly, we show below that the same SV40 virus induces in primary rat and mouse cells from the same tissue of origin, very different neoplastic clones with individually different morphologies and growth rates.
3. *Virus-induced tumors and neoplastic clones without viral proteins and genes*. In searching for a viral role in carcinogenesis over 20 studies have found no viral T-antigen and no viral DNA in neoplastic clones induced by Polyoma virus [10, 47–50] and SV40 [2, 11, 12, 27, 51–59]. These results were initially described as “perplexing exceptions” in an influential review of the virus-cancer theory [2]. Subsequently, it was found that tumors induced in mice with transcriptionally controllable transgenic SV40 T-antigens do not revert to normal, when their T-antigens are switched off [33]. Moreover, there are recurrent reports that tumors from mice with transgenic viral T-antigens are free of T-antigen or viral genes or both [34, 35, 60–62]. One such study from our lab found that four of nine tumors of mice with transgenic SV40-T-antigen genes lacked viral T-antigens and mRNAs of T-antigen altogether [35]. Another study even found “increased oncogenicity” after the loss of T-antigen [62]. In addition tumors of transgenic mice were found to be heterogeneous, containing both T-antigen-negative and positive cells by others and us [35, 63, 64] (see also Results). It follows that viral genes and proteins are not necessary to maintain neoplastic phenotypes such as cancer-specific reproductive autonomy and immortality. This explains, why SV40 and polyoma viruses with conditional transforming genes, which would allow neoplastic clones to revert to normal cells under non-permissive conditions, were never found - despite enormous efforts [2, 3, 33, 46, 65–68].
4. *Viruses induce preneoplastic aneuploidy and neoplasias with abnormal karyotypes and transcriptomes.* Unexpectedly, it was discovered in 1962 that SV40 induces proliferation of human cells with heterogeneous aneuploidies and abnormal cell morphologies within days after infection [39, 40, 51, 69–73]. This discovery was immediately seen as a breakthrough in cancer research. Accordingly Shein and Enders wrote in 1962: “Accelerated growth, abnormal growth pattern, and chromosomal aberrations exhibited by E cells (SV40-transformed human epithelial cells) are characteristics commonly associated with rapidly growing tumors and with “continuous” lines of cells in culture.” [39]. Subsequently, abnormal karyotypes and / or transcriptomes and phenotypes were found in human cell lines “immortalized” by virus or by transfection with genes of viral T-antigens [12–18, 25, 42–44, 74–86]. Abnormal karyotypes and / or transcriptomes were also found in neoplastic clones arising from cultures of SV40 and Polyoma virus-infected primary hamster [9, 10, 27, 87], rat [3, 12, 41, 88] and mouse cells [37, 38, 89]. And were also found in tumors induced by Polyoma virus in mice [30] and by transgenic SV40 T-antigens in rats [90] and in mice [35, 37, 38]. Since a majority of clonal tumors from transgenic mice were T-antigen-free, we concluded in 2010 that these clonal karyotypes have neoplastic function [35]. Surprisingly, none of the currently prevailing virus-cancer theories cited above [4–7] mentions the abnormal karyotypes and / or transcriptomes of SV40 and Polyoma virus-infected or transfected preneoplastic cells and of the immortal neoplastic clones and tumors that arise from such cells.

### Alternative theory of virus-induced neoplastic transformation based on karyotype alteration

Given these four unexplained characteristics of viral carcinogenesis, particularly the preneoplastic aneuploidy of virus-infected cells and the individual abnormal karyotypes and the immortality of virus-induced neoplastic clones or tumors, we speculated that viral carcinogenesis might be a form of speciation [91–95] - much like non-viral carcinogenesis [96–99].

**Fig. 1:**
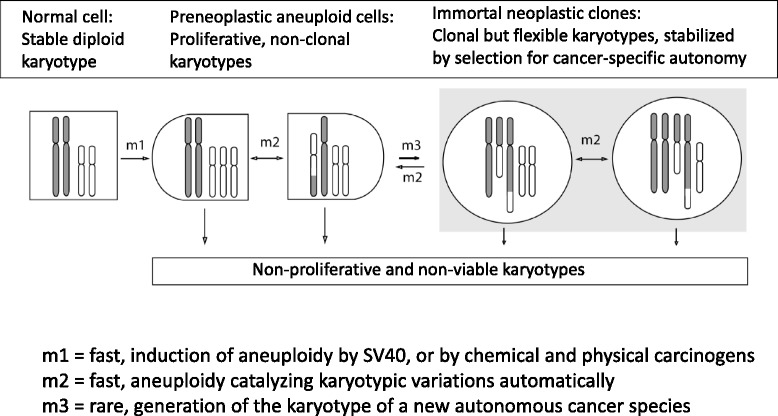
Karyotypic theory of SV40 virus-induced neoplastic transformation. The karyotypic theory proposes that SV40 initiates carcinogenesis indirectly by inducing in infected cells preneoplastic aneuploidies at high rates (m1, in Fig. 1). Since aneuploidy destabilizes the karyotype by unbalancing thousands of genes, it catalyzes chain reactions of karyotypic and transcriptomic evolutions, also at high rates (m2, in Fig. 1). Eventually rare karyotypes evolve that encode cancer-specific autonomy of growth, at very low rates (m3, Fig. 1). The low probability of forming new autonomous cancer-species by random karyotypic and transcriptomic variations predicts the individuality of cancers. Although cancer karyotypes are congenitally aneuploid and thus unstable, they are stabilized or immortalized by selection for variants with cancer-specific autonomy. Owing to these inherent variations cancer karyotypes are heterogeneous within clonal margins (shaded in Fig. 1) [100–102]. The resulting spreads of quasi-clonal karyotypes are defined by ‘karyotype arrays’ in which multiple individual karyotypes of the same cancers are compared (shown below in Figs. 5, 6, 7, 11, 12, 13, 16 and 17)

Accordingly, we propose that SV40 induces cancer indirectly by inducing preneoplastic aneuploidy at high rates (m1, in Fig. 1). Since aneuploidy destabilizes the karyotype by unbalancing thousands of genes, it catalyzes chain reactions of karyotypic and transcriptomic evolutions, also at high rates (m2, in Fig. 1). Eventually rare karyotypes evolve that encode cancer-specific autonomy of growth, at very low rates (m3, Fig. 1). The low probability of forming new autonomous cancer-species by random karyotypic and transcriptomic variations predicts individual and clonal cancers. Although cancer karyotypes are congenitally aneuploid and thus variable, they are stabilized or immortalized by selections for variants with cancer-specific autonomy. Owing to these inherent variations cancer karyotypes are heterogeneous within clonal margins (shaded in Fig. 1) [100–102]. The resulting spread of quasi-clonal karyotypes is illustrated below in ‘karyotype arrays’ in which multiple individual karyotypes of the same cancer are compared (see Results).

In sum this karyotypic theory proposes that SV40 and Polyoma viruses generate clonal immortal cancers indirectly by inducing aneuploidy, which catalyzes the rare evolution of new cancer-causing karyotypes.

To test the karyotypic theory of viral carcinogenesis, we analyzed here the karyotypes and phenotypes of virus-infected (1) human mesothelial, (2) rat lung, and (3) mouse embryo cells from the time of infection to the origins of immortal neoplastic clones. As we show below, we found in each of these three systems, (1) preneoplastic heterogeneous aneuploidies, (2) neoplastic clones with individual clonal karyotypes and phenotypes, which arose from one in 10,000 infected cells and survived over 200 generations, (3) spontaneous and drug-induced phenotypic variations of neoplastic clones that correlated 1-to-1 with karyotypic variations, but no consistent correlations with SV40 genes.

Since all 14 virus-induced neoplastic clones tested contained individual quasi-clonal karyotypes, we concluded that these karyotypes generate the complex individual phenotypes and transcriptomes of neoplastic clones, rather than common viral genes, which are not present in dozens of neoplastic clones (Results and Background). It would follow that the carcinogenic action of SV40 virus is indirect, as an initiator of the evolution of cancer causing karyotypes.

## Results and discussion

In the following we have tested the karyotypic theory of viral transformation in SV40-infected primary human (I), rat (II) and mouse cells (III).

### I. Transformation of human cells by SV40

To test the theory that SV40 transforms normal human cells to neoplastic cells indirectly by inducing preneoplastic aneuploidy (see Fig. 1), we have used an established system of viral transformation described by Bocchetta et al. in 2000 [21]. In this system SV40 induces enhanced growth and preneoplastic transformation in primary human mesothelial cells within two weeks after infection and rare neoplastic clones from less than one of 10,000 infected cells two to three months after infection [21].

### Phenotypes and karyotypes of SV40-infected preneoplastic human mesothelial cells

#### Phenotypes of SV40-infected preneoplastic cells

**Fig. 2:**
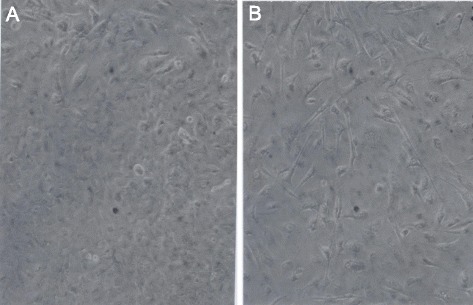
Phenotypes of human mesothelial cells three weeks after infection by SV40 compared to an uninfected control. A subconfluent culture of human mesothelial cells was infected with SV40 at a multiplicity of 10. In parallel an uninfected culture was maintained under the same conditions (see text). **a** A 120X magnification of the infected culture three weeks after infection shows highly increased cell density and “pre-transformed” cell morphologies [13], compared to the uninfected control shown in (**b**). It follows that SV40 transforms the morphology and raises the density of cultures of mesothelial cells shortly after infection

To determine the phenotypes of preneoplastic SV40-infected mesothelial cells, we infected a sub-confluent culture of primary mesothelial cells with SV40 at a multiplicity of infection of 10 and kept an un-infected control under the same conditions. Comparison of infected and uninfected cells by light microscopy two weeks after infection indicated that the infected cells had formed dense multilayers of polymorphic cells with rounded and oval shapes, as described by Bocchetta et al. [21] and shown in Fig. 2a. In parallel with enhanced growth, the infected mesothelial culture also shed a relatively large minority of infected cells, which would not reattach to a new culture dish. This SV40-induced enhanced proliferation and degeneration of cells also confirms previous observations of Bochetta et al. and many others [8, 13, 21, 31, 39, 51]. By contrast, the uninfected culture consisted of partly spindle-shaped and partly rounded cells and barely reached confluence under the same conditions (Fig. 2b).

#### Karyotypes of SV40-infected preneoplastic cells

To test for the predicted aneuploidies of preneoplastic cells (Fig. 1), we analyzed the karyotypes of the SV40-infected human mesothelial cells shown in Fig. 2 one and two months after infection. As shown in Table 1, we found that 65 % (13 of 20) of the infected human cells carried heterogeneous, near-diploid aneuploidies at one month after infection. At two months after infection 95 % (19 per 20) of these cells carried heterogeneous aneuploidies, of which three had near tetraploid karyotypes, as is shown in Table 2. By contrast, the uninfected mesothelial controls could not even be karyotyped for lack of mitoses.

**Table 1:** Karyotypes of mesothelial cells 1 month after infection with SV40: 13 aneuploid per 20

Karyotypes	1	2	3	4	5	6	7	8	9	10	11	12	13
Total no of chromosomes	47	45	45	47	49	45	45	47	47	44	48	46	42
Chromosomes													
1	2	2	2	2	2	1	2	2	2	2	2	2	2
2	2	2	2	2	2	2	2	2	2	3	2	2	2
3	2	2	2	2	2	2	2	2	2	2	2	2	1
4	2	2	2	2	2	2	2	2	2	2	2	2	2
5	2	2	2	2	2	2	2	3	3	2	2	3	2
6	2	2	2	2	2	2	2	2	2	2	2	2	1
7	2	2	2	2	2	2	1	2	2	2	2	2	2
8	2	2	2	2	2	2	2	2	2	2	2	2	1
9	2	2	2	2	2	2	2	2	2	2	3	2	2
10	2	2	2	2	2	2	2	2	2	2	2	2	2
11	2	2	2	2	2	2	2	2	2	2	2	1	2
12	2	2	2	2	2	2	2	2	2	2	2	2	2
13	2	2	2	2	2	2	2	2	2	2	2	3	2
14	2	2	2	2	2	2	2	2	2	2	2	2	2
15	2	2	2	3	2	2	2	2	2	2	2	2	2
16	2	2	2	2	2	2	2	2	2	1	2	2	2
17	2	2	2	2	3	2	2	2	2	1	2	2	2
18	2	2	2	2	2	2	2	2	2	2	2	1	2
19	2	2	2	2	3	2	2	2	2	2	2	2	2
20	2	2	2	2	2	2	2	2	2	1	3	2	2
21	2	1	2	2	3	2	1	2	2	1	2	2	2
22	2	2	2	2	2	2	2	2	2	2	2	2	2
X	2	2	1	2	2	2	2	2	2	2	2	2	1
Y	0	0	0	0	0	0	0	0	0	0	0	0	0
der(7;21)?	0	0	0	0	0	0	1	0	0	0	0	0	0
der(7)	1	0	0	0	0	0	0	0	0	0	0	0	0
i(21q)	0	0	0	0	0	0	0	0	0	1	0	0	0

**Table 2:** Karyotypes of mesothelial cells two months after infection with SV40: 19 aneuploid per 20

Karyotypes	1	2	3	4	5	6	7	8	9	10	11	12	13	14	15	16	17	18	19
Total no of chromosomes	45	46	46	43	46	46	47	47	46	47	47	47	47	48	49	60	75	75	83
Chromosomes																			
1	2	2	2	2	2	2	2	2	1	1	2	2	2	2	2	2	3	4	4
2	1	2	2	2	2	2	2	2	2	2	2	2	2	2	2	3	1	4	3
3	2	2	2	2	2	2	2	2	2	2	3	2	2	2	2	2	3	4	3
4	2	2	2	2	2	2	2	2	2	2	2	2	2	3	2	4	2	3	4
5	2	2	2	2	3	2	3	2	2	1	2	2	3	2	3	1	5	3	4
6	1	2	2	2	2	2	2	3	2	2	2	2	2	3	2	2	3	4	2
7	2	2	2	2	2	2	2	2	2	2	2	2	2	2	2	2	4	2	3
8	2	2	2	2	2	2	2	2	2	2	2	2	2	2	2	3	3	2	3
9	2	2	2	2	2	2	2	2	1	2	2	2	2	2	2	3	3	2	3
10	2	1	2	2	2	2	2	2	2	3	2	2	2	2	2	2	4	4	4
11	2	2	2	2	2	2	2	2	2	2	2	2	2	2	2	2	3	3	3
12	2	2	2	2	2	2	2	2	2	2	2	2	2	2	2	3	4	3	4
13	2	2	2	2	2	2	2	2	2	2	2	2	2	2	2	2	4	3	3
14	2	2	1	1	1	2	2	2	2	2	2	2	2	2	2	2	4	4	4
15	2	2	2	1	2	1	2	2	2	1	2	2	2	2	2	4	4	3	4
16	2	2	2	1	2	2	2	2	2	2	2	2	2	2	2	4	2	3	2
17	2	2	2	2	1	2	2	2	2	2	2	2	2	2	2	1	4	4	3
18	2	2	2	2	2	2	2	2	2	2	2	2	1	3	2	2	3	2	2
19	2	2	2	2	2	2	2	2	1	1	2	2	2	1	3	2	3	3	4
20	2	1	2	2	2	2	2	2	2	2	2	2	2	2	3	4	4	4	4
21	2	2	2	2	2	2	2	2	2	2	2	2	2	2	2	1	2	3	3
22	2	2	2	2	2	2	2	2	2	2	2	2	2	2	2	4	2	3	2
X	2	2	2	2	2	2	2	2	2	2	2	2	2	2	2	1	3	3	4
Y	0	0	0	0	0	0	0	0	0	0	0	0	0	0	0	0	0	0	0
der(1q)	0	0	0	0	0	0	0	0	1	1	0	0	0	0	0	0	0	0	0
der(18) long	0	0	0	0	0	0	0	0	0	0	0	0	1	0	0	0	0	1	0
der(9;6;9;6)	0	0	0	0	0	0	0	0	0	0	0	0	0	0	0	0	0	0	1
der(17;18)	0	0	0	0	0	0	0	0	0	0	0	0	0	0	0	0	0	0	1
der(22;7)	0	0	0	0	0	0	0	0	0	0	0	0	0	0	0	0	0	0	1
der(16;8;3)	0	0	0	0	0	0	0	0	0	0	0	0	0	0	0	0	0	0	1
der(8;3)	0	0	0	0	0	0	0	0	0	0	0	0	0	0	0	0	0	0	1
der(22;16)	0	0	0	0	0	0	0	0	0	0	0	0	0	0	0	0	0	0	1
min(3?)	0	0	0	0	0	0	0	0	0	0	0	0	0	0	0	0	0	0	1
der(8)	0	0	0	0	0	0	0	0	0	0	0	0	0	0	0	0	0	0	1
der(1;17)	0	0	0	0	0	0	0	0	0	0	0	0	0	0	0	1	0	0	0
der(17;12)	0	0	0	0	0	0	0	0	0	0	0	0	0	0	0	1	0	0	0
der(22) small	0	0	0	0	0	0	0	0	0	0	0	0	0	0	0	1	0	0	0
min(19)	0	0	0	0	0	0	0	0	0	0	0	0	0	0	0	1	0	0	0
der(19q)	0	0	0	0	0	0	0	0	1	0	0	0	0	0	0	0	0	0	0
der(9;13?;9)	0	0	0	0	0	0	0	0	1	0	0	0	0	0	0	0	0	0	0
der(16q)	0	0	0	0	0	0	0	0	0	0	0	0	0	0	0	0	2	0	0
der(19;14)	0	0	0	0	0	0	0	0	0	1	0	0	0	0	0	0	0	0	0
der(1)	0	0	0	0	0	0	0	0	0	1	0	0	0	0	0	0	0	0	0
dic(5;15)	0	0	0	0	0	0	0	0	0	1	0	0	0	0	0	0	0	0	0
i(14q)	0	0	1	0	0	0	0	0	0	0	0	0	0	0	0	0	0	0	0
dic(2;6)	1	0	0	0	0	0	0	0	0	0	0	0	0	0	0	0	0	0	0
der(13) small	0	0	0	0	0	0	0	0	0	0	0	1	0	0	0	0	0	0	0
der(15;3)	0	0	0	0	0	1	0	0	0	0	0	0	0	0	0	0	0	0	0
der(12;9)	0	0	0	0	0	0	0	0	0	0	0	0	0	0	0	0	0	1	0
der(10q)	0	1	0	0	0	0	0	0	0	0	0	0	0	0	0	0	0	0	0
der(20) long	0	1	0	0	0	0	0	0	0	0	0	0	0	0	0	0	0	0	0
der(17)	0	0	0	0	1	0	0	0	0	0	0	0	0	0	0	0	0	0	0

We conclude from the high percentages of aneuploidization of cells early after infection, that the virus induces aneuploidy directly, probably by the viral T-antigen. Accordingly, T-antigen induces aneuploidy either by binding randomly to chromosomes, or to specific mitosis proteins as suggested by others [85, 86].

With these results we confirmed that SV40 alters the phenotypes, the growth rate, the karyotype and also kills a fraction of the infected human cells within weeks after infection. These results are thus compatible with the theory that SV40-induced aneuploidies with proliferative phenotypes enhance growth and alter cellular phenotypes. Simultaneously virus-induced aneuploidies with lethal phenotypes would kill cells (Fig. 1) in addition to lytic infections [3].

### Phenotypes and karyotypes of two neoplastic lines from SV40-infected mesothelial cells

To test the theory that individual clonal karyotypes, rather than viral genes, generate and immortalize new neoplastic clones with individual phenotypes (see Fig. 1), we have analyzed the phenotypes and karyotypes of two immortal lines derived from SV40-infected mesothelial cells. These lines or clones have been isolated from infected mesothelial cells and termed F1 and F4 by Bocchetta et al. [21]. Bocchetta et al. also proved that these lines are immortal by demonstrating that the lines survived over 200 generations in culture. Neoplastic lines are designated “immortal”, “permanent” or “continuous” in the literature, if they have survived over 100 generations in culture or as transplants in animals [5, 7, 13, 14, 17, 89, 103]. By contrast, normal cells fail to survive over 50 generations in these conditions [104].

#### Phenotype of the neoplastic F1 line

**Fig. 3:**
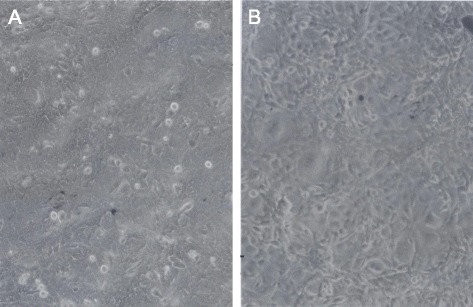
Cell morphologies of the immortal cell lines F1 and F4 derived from SV40-infected human mesothelial cells. The karyotypic cancer theory predicts that neoplastic karyotypes encode the individual phenotypes of SV40-transformed neoplastic clones. To test this prediction we have compared at 120X magnifications cultures of the immortal neoplastic cell lines F1 and F4, which arose from SV40-infected human mesothelial cells. It can be seen in the micrographs shown in (**a**) that the F1 line formed a dense monolayer of cells and in (**b**) that the F4 line formed a multilayer of cells. The micrographs also show that both lines consisted of round to oval polymorphic cells, and that the F4-cells were on average larger than the F1 cells. This result revealed phenotypic cellular similarity but sociological dissimilarity of the two cell lines

As can be seen in Fig. 3a, the F1 line formed a dense, mostly single-layered culture of cells with rather uniform, F1-specific round to oval morphologies, which differed from the heterogeneous cell morphologies of the preneoplastic mass culture, shown in Fig. 2a. This result supports the theory that the F1 cells are encoded by a clonal F1-specific genotype, which would be a karyotype in the light of our theory.

#### ‘Karyotype array’ of the neoplastic F1 line

**Fig. 4:**
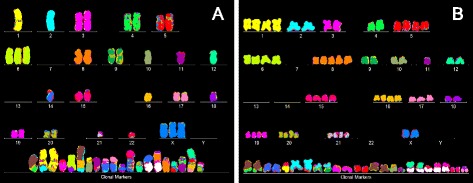
Karyotypes of the immortal cell lines F1 and F4 derived from a common culture of SV40-infected human mesothelial cells. To test, whether the karyotypes of F1and F4 would explain the individual but related phenotypes of F1 and F4, we compared their karyotypes. The karyotypes were prepared from metaphase chromosomes stained with chromosome-specific fluorescent colors following published procedures (Methods). As shown in (**a**), F1 has a hyper-diploid, aneuploid karyotype with 56 including 36 normal and 20 marker chromosomes. The karyotype shown in (**b**) indicates that F4 has a hypo-tetraploid karyotype with 81 including 56 normal and 25 marker chromosomes. However, a close comparison of the copy numbers of the ten F1-chromosomes, 2, 5, 10, 11, 12, 15, 18, 19, 20 and of one shared marker chromosome (the first on the list of marker chromosomes), with their F4-counterparts reveals that the F1-copy numbers are exactly duplicated in F4. This suggests that F4 probably originated from F1 by some form of karyotype duplication (see text)

Next we asked, whether the F1 line has the predicted F1-specific clonal karyotype (see Fig. 1). For this purpose we have stained metaphase chromosomes of F1 with chromosome-specific color-coded DNA probes and arranged the chromosomes based on these colors into a conventional human karyotype with a computer-assisted microscope, following published procedures (Methods) [101, 102]. The resulting karyotype shows in Fig. 4a that F1 has indeed a specific, abnormal human karyotype with a total number of 54 chromosomes, which include 20 cancer-specific hybrid or marker chromosomes, instead of the normal 46 human chromosomes.

But to determine, whether the F1-karyotype shown in Fig. 4a is indeed clonal, multiple karyotypes of the same putative clone must be compared. To meet this end, we have used the recently developed ‘karyotype arrays,’ which are designed to compare multiple karyotypes of the same clone [101, 102]. Karyotype-arrays are three-dimensional tables of typically 20 karyotypes, which list the chromosome numbers of each karyotype on the x-axis, the copy numbers of each chromosome on the y-axis, and the number of karyotypes arrayed on the z-axis. Such arrays are specifically useful to define the degree of clonality and individuality of the inherently flexible karyotypes of cancers (see Fig. 1) for two reasons: Firstly, they reveal at a glance the degree of clonality, or lack of it, by parallel lines that are formed by the chromosomes of karyotypes with the same copy numbers. Secondly, arrays also reveal at a glance the individuality of clones in comparison with others, by forming individual patterns of clonal chromosome copy numbers, which are readily distinguishable from those of other clones.

**Fig. 5:**
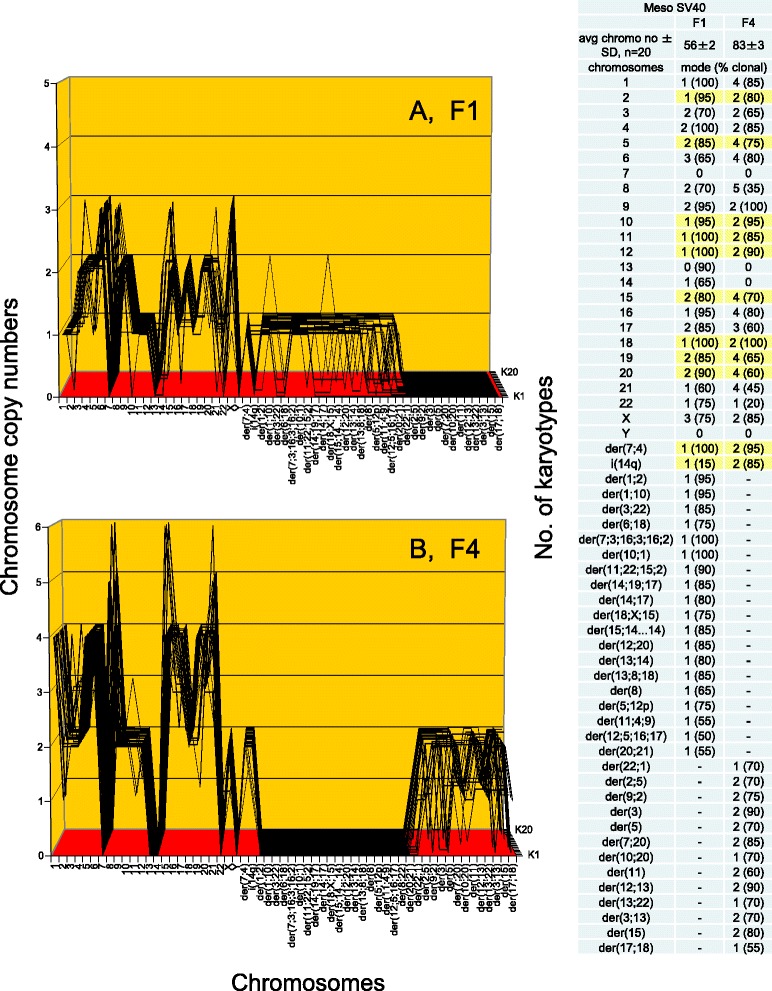
Comparison of the karyotype arrays of the immortal F1 and F4 lines from SV40-infected human mesothelial cells*.* Karyotype arrays are three-dimensional tables of 20 karyotypes, which list the chromosome numbers of each karyotype on the x-axis, the copy numbers of each chromosome on the y-axis, and the number of karyotypes arrayed on the z-axis, as detailed in the text (Section I, *Phenotypes and karyotypes of neoplastic clones from SV40-infected mesothelial cells*). **a** and the attached table shows that the F1 line is hyper-diploid, consisting of 56 chromosomes that are 70 % to 100 % clonal. Accordingly the F1-chromosomes formed a quasi-clonal F1-array, which defines the F1 line. The non-clonal fraction of chromosomes included several partially clonal and several non-clonal marker chromosomes, indicative of ongoing karyotypic variation (see Fig. 1). **b** shows the F4 line is hypo-tetraploid consisting of 83 chromosomes which are 60–100 % clonal. Accordingly the F4-chromosomes formed a quasi-clonal F4-array, which is distinct from, but visibly related to that of the F1 line. The attached tables indicate that the differences between F1 and F4 include 19 F1-specific and 13 F4-specific clonal marker chromosomes. The tables also indicate that the copy numbers of 10 intact and one F4 marker chromosomes were exact duplications of the copy numbers of the corresponding F1-chromosomes (marked yellow in Fig. 5). In addition F1 and F4 shared two nullisomies of chromosomes 7 and 13. Moreover, several F1-chromosomes with non-duplicated copy numbers in F4 were increased in F4, but not exactly two-fold. This result thus indicates that the F4 line is a descendant of the F1 line generated by some form of tetraploidization (see text), rather than an independent clone

As shown in Fig. 5a and the attached table, the karyotype array of F1 consists of an average number of 56 chromosomes (confirming Fig. 4a), which are 70 to 100 % clonal. Accordingly, the 56 F1-chromosomes formed the quasi-clonal, F1-specific karyotype array that is shown in Fig. 5a. In addition the F1-array also showed the 0-30 % of F1-chromosomes with non-clonal copy numbers, which reflect the inherent flexibility of cancer karyotypes (see above, Fig. 1).

In sum, we have demonstrated that the immortal F1 line has an individual (compared to normal) clonal karyotype and phenotype. The clonality and individuality of the F1-karyotype array confirm the prediction of the karyotypic theory that this karyotype has generated and since maintained the phenotype of the F1 line for over 200 generations.

The individuality and high complexity of the transcriptomes of SV40-transformed human neoplastic clones, which consist of hundreds of abnormally expressed mRNAs [43, 44], directly confirm the view that complex individual karyotypes, rather than common viral genes, encode the complex individual phenotypes of SV40-induced neoplastic clones.

We conclude that the F1 karyotype is either sufficient for neoplastic transformation or is necessary and possibly also dependent SV40 genes. If SV40-transformation were indeed dependent on SV40-genes, those genes would have to be present in all viral tumors and neoplastic clones, which we tested below (See, *Non-correlations between SV40 T-antigen and the cells of the immortal clones F1 and F4*, and Background).

### Karyotypic variations of the neoplastic F1 line generate phenotypically distinct sub-clones

#### Variation of the F1 line to the phenotypically distinct F4 line

In an effort to confirm the predicted individuality of new neoplastic clones like F1, we compared the F1 line with a second immortal neoplastic line from SV40-infected human mesothelial cells, which was also isolated by Bocchetta et al. and named the F4 line [21].

##### The F4 phenotype

To determine, whether F4 has an individual clonal phenotype, as predicted for an independent neoplastic clone by the karyotypic theory, we set up parallel cultures of F4 and F1, in which F1 served as a standard of comparison. As can be seen in Fig. 3b, the F4 line formed a multi-layered culture, which was distinct from the mostly mono-layered culture of F1 shown in Fig. 3a. But, the comparison also showed that both clones consist of cells with round to oval morphologies. These results thus indicated that the two lines are distinct sociologically, but have very similar cellular morphologies. According to our theory that cancer karyotypes encode cancer phenotypes, this result predicts that the karyotypes of F1 and F4 are related, yet distinct. This predicted karyotypic relationship of F1 and F4 was tested next.

##### The F4 karyotype

Comparisons of the karyotypes and the karyotype arrays of F1 and F4 are shown in Figs. 4 and 5. As can be seen in Figs. 4b and 5b and the attached tables, the F4 line has a hypo-tetraploid karyotype with an average number of 83 chromosomes. The copy numbers of these chromosomes were 60-100 % clonal. Accordingly the F4-chromosomes formed a quasi-clonal F4-specific karyotype array, which is different from, but is also similar to that of the F1 line with only 56 chromosomes (compare Fig. 5a and b).

Accordingly, a close examination of the arrays of F1 and F4 revealed that 11 F4-chromosomes including one shared marker chromosome are exact duplications of the copy numbers of the corresponding F1-chromosomes (marked yellow in the table of Fig. 5). In addition the comparison shows that F1 and F4 also share two clonal nullisomies of chromosomes 7 and 13. Furthermore, several F4-chromosomes with non-duplicated copy numbers, compared to F1, were also increased in F4, but not exactly two-fold.

As a result the karyotype of F4 can be said to be an approximately two-fold amplification or tetraploidization of that of F1. Obviously this tetraploidization occurred together with the gain of 13 F4-specific markers and the loss of 19 F1-specific marker chromosomes, as can be seen in Figs. 4 and 5. We deduce from these karyological data that the F4 clone branched off from the original F1 clone by an approximate tetraploidization, including losses of F1-specific and gains of new F4-specific marker chromosomes. It is also consistent with our results that F4 and F1 derived from a common unknown, but near-diploid F1-like precursor of F1 and F4, and that each clone subsequently diverged individually.

Thus the F4 line is a variant of the F1 line, rather than an independent clone as initially expected [21]. This karyotypic relationship between F1 and F4 explains directly their phenotypic relationship that is shown in Fig. 3.

To test the view that SV40-induced neoplastic clones undergo spontaneous variations, we searched for additional, experimentally controllable variations of cancer phenotypes by karyotypic variations. For this purpose we studied next karyotype variations correlating with experimentally induced drug-resistance of F1 and F4.

#### Variation of F1 to a puromycin-resistant F1-variant

**Fig. 6:**
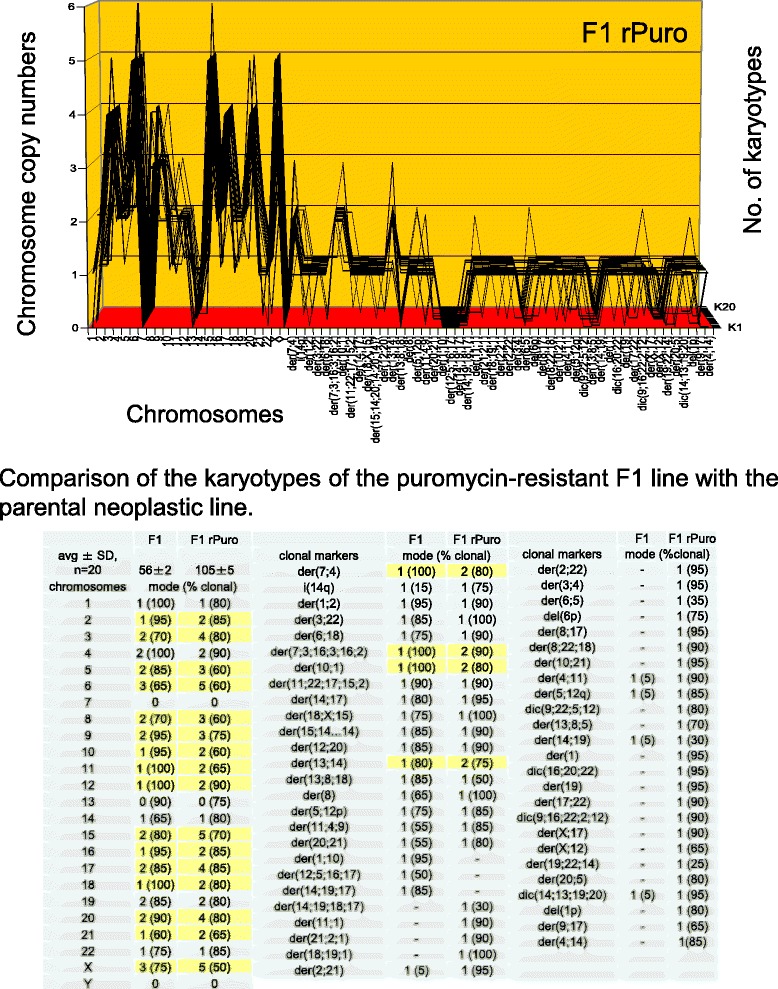
Comparison of the karyotype array of the F1 line with that of a puromycin-resistant derivative. To determine whether acquisition of resistance to puromycin was based on karyotypic variation, the karyotype array of a puromycin-resistant variant of F1was compared to that of the parental F1 clone. The array of the drug-resistant F1 shows a hyper-tetraploid karyotype consisting of 105 chromosomes that were 45–100 % clonal. Accordingly these chromosomes formed a quasi-clonal, resistant F1-specific array, which is distinct from, but visibly related to that of the parental F1 line, shown in Fig. 5a. Quantitative comparison of the chromosome copy numbers of the two F1 variants shown in the table of Fig. 6 revealed obvious similarities: 14 F1-chromsomes were exactly duplicated in the resistant variant and six others were increased approximately two-fold. It follows that the puromycin-resistant variant of F1 arose from the parental F1 line by an approximate tetraploidization; similar to how the above described near tetraploid F4 arose from the near diploid F1. This event was also associated with the acquisition of 29 new resistance-specific clonal marker chromosomes, and with the loss of three parental clonal marker chromosomes

A puromycin-resistant variant of the F1 line was generated by selection of survivors at increasing concentrations of puromycin up to 2 μg per ml medium following published procedures [97, 102, 105]. The karyotype of the puromycin-resistant F1 was then compared with that of the parental F1 based on the karyotype arrays shown in Figs. 5a and 6.

As can be seen in Fig. 6, the puromycin-resistant F1 line has a hyper-tetraploid karyotype with an average number of 105 chromosomes, which are 45–100 % clonal. Accordingly, these 105 chromosomes formed an individual quasi-clonal karyotype array that is different from, but also similar to that of the hyper-diploid parental F1 line, shown above in Fig. 5a.

Moreover, a close comparison of the chromosome copy numbers of the puromycin-resistant F1-variant with the parental F1 line revealed that the copy numbers of 14 F1-chromosomes are exactly duplicated and six are increased to lesser or higher degrees in the puromycin-resistant F1 variant, compared to the parental clone. These duplications and concomitant near two-fold increases of chromosome copy numbers are marked yellow in the table of Fig. 6.

Thus the puromycin-resistant F1 is indeed a karyotypic variant of F1, rather than an independent clone. The underlying karyotypic variation was once more based on an approximate tetraploidization of the F1 karyotype (as in the variation from F1 to F4 described above). This tetraploidization coincided with the generation of 29 new resistance-specific marker chromosomes, the loss of three parental marker chromosomes and changed copy numbers of some shared non-duplicated chromosomes, marked yellow in the table of Fig. 6. The karyotypic variation that sets apart F1 from the puromycin-resistant variant provides thus a consistent explanation for the corresponding phenotypic variation, namely the acquisition of the new puromycin-resistant phenotype.

#### Variation of F4 to a puromycin-resistant F4-variant

In the following the karyotype of F4, itself a variant of F1 (see Fig. 5 above), was compared to the karyotype of a variant of F4 that was resistant to puromycin at 2 μg per ml medium. The puromycin-resistant F4 line was prepared as described for the puromycin-resistant variant of F1 above.

**Fig. 7:**
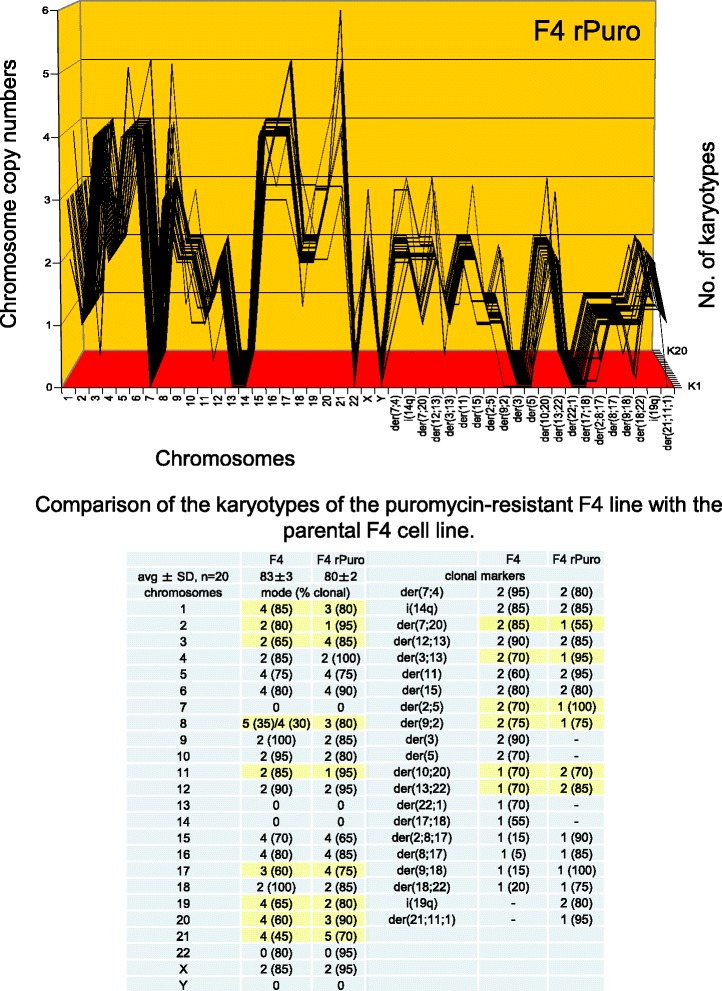
Comparison of the karyotype array of the F4 line with that of a puromycin-resistant derivative*.* To determine whether resistance to puromycin of the F4 line was acquired by karyotypic variation, as was found in Fig. 6, the karyotype array of a puromycin-resistant F4 variant was compared to that of the parental F4 line. The array of the drug-resistant F4 shows a hypo-tetraploid karyotype consisting of 80 chromosomes that were 55–100 % clonal. Accordingly, these chromosomes formed a quasi-clonal array, which is distinct from, but visibly related to from the parental F4 line, shown in Fig. 5b. Specifically, the puromycin-resistant F4 variant differs from the parental line in the copy numbers of 15 of their 34-shared chromosomes (marked yellow in Fig. 7), the gain of two resistance-specific marker chromosomes and the loss of four parental marker chromosomes. We conclude that the F4 line acquired resistance to puromycin by karyotypic variation, as was the case with the puromycin-resistant variant of the F1 clone described in Fig. 6

As shown in Fig. 7 and the attached table, the puromycin-resistant F4 has a hypo-tetraploid karyotype with an average number of 80 chromosomes, which were 70–100 % clonal. Accordingly the chromosomes of drug-resistant F4 formed a clonal puromycin-resistant F4-specific karyotype array. This drug-resistant-F4 array is visibly related to, but also different from that of the parental F4 array, shown in Fig. 5b. Close comparisons of the two arrays indicate that the resistant F4 clone shared with the parental F4 clone 23 chromosomes with the same copy numbers including three rare nullisomies. The comparison further shows that the resistant clone differed from the parental clone randomly in the copy numbers of 13 intact chromosomes, the gain of two resistance-specific marker chromosomes and the loss of four parental marker chromosomes. Hence, the karyotypic variation that sets apart the puromycin-resistant F4-variant from the parental F4 is again consistent with the acquisition of puromycin-resistance by karyotypic variation, rather than being an independent clone.

In sum, all three examples of phenotypic variations of the original neoplastic F1 line support the theory that karyotype variations of neoplastic clones cause phenotype variations.

### Karyotypic variation and origination: two distinct mechanisms generating new neoplastic clones

As a result of our comparative karyotypic analyses of immortal neoplastic clones derived from SV40-infected human mesothelial cells, we have now before us two distinct mechanisms that generate new neoplastic clones with distinct individual phenotypes:

1. The independent generation of new neoplastic clones with new individual clonal karyotypes and phenotypes from preneoplastic SV40-infected, aneuploid cells at very low rates (i.e., m3 in Fig. 1). Examples are the F1 clone from human mesothelial cells and 10 other neoplastic clones from SV40-infected rat and mouse cells described below (Sections II and III).
2. The generation of new neoplastic clones by variations of the karyotypes of existing neoplastic clones at rather high rates (i.e., m2 in Fig. 1). Examples are the three variants or sub-clones of the mesothelial F1 stem line described in Figs. 5, 6 and 7. We have described previously additional examples of karyotypic variants with new phenotypes derived from other established primary cancers, such as drug-resistant variants [105, 106] and metastatic variants [97, 107].

It may be argued, however, that the complex new individual karyotypes and transcriptomes of neoplastic clones are not sufficient to generate, maintain and vary their neoplastic phenotypes and that SV40-genes are also necessary. To test this possibility, we have asked whether viral proteins are consistently present in the virus-induced neoplastic line F1 and its derivative variants.

### Non-correlations between SV40 T-antigen and the cells of the immortal lines F1 and F4

Our evidence that specific karyotypes generate and maintain the phenotypes of SV40 virus-induced neoplastic clones and of their variants raises the question, whether the initiating SV40 has any direct etiological role in maintaining and varying neoplastic clones. If so, all virus-induced neoplastic clones should contain SV40 genes, particularly the genes of the viral T-antigens [4–7].

**Fig. 8:**
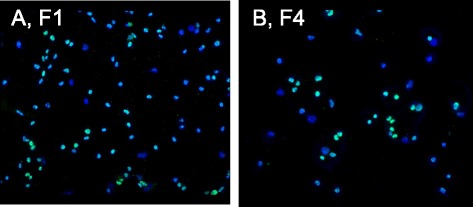
Non-correlations between SV40 T-antigen and the cells of the immortal clones F1 and F4. To answer the question whether clonal cancer karyotypes are sufficient to generate and maintain neoplastic clones or are also dependent on T-antigen, we analyzed the immortal neoplastic clones F1 and F4 for the presence of viral T- antigen. For this purpose F1 and F4 cell cultures were reacted with mouse anti-T-antigen antibodies-linked to a green fluorescent dye and counter-stained with the blue fluorescent DNA dye, ‘DAPI,’ to detect nuclear DNA irrespective of T-antigen (Methods). **a** shows that about 30 % of the cells of the F1 line were T-antigen negative, while the remaining 70 % were heterogeneous for T-antigen expression ranging from very low to relatively high levels. **b** shows that about 50 % of the cells of F4 were T-antigen negative, whereas the rest of the cells were heterogeneous ranging from very low to relatively high T-antigen levels, similar to the F1 culture. We conclude that T-antigen is not necessary to maintain neoplastic transformation of F1 and F4 lines

To answer this question, we have reacted cultures of the immortal F1 and F4 clones with primary mouse antibodies against the T-antigen of SV40 and green-fluorescent secondary goat antibodies following the manufacturer’s protocol (Abcam Inc., Cambridge, MA). In addition we have also counter-stained the same cultures with the blue-fluorescent DNA-specific dye Diamidino-2-Phenylindole (DAPI) to detect all cells of the F1 and F4 cultures, independent of the presence of T-antigen. As can be seen in Fig. 8a, about 30 % of the F1-cells were negative for T-antigen. In the remaining cells the concentrations of T-antigen ranged from relatively low to high. Similarly, Fig. 8b shows that about 50 % of the F4-cells were negative for T-antigen. In the T-antigen-positive F4 cells the concentrations of the antigen also ranged from relatively low to high. Prior studies of negative and heterogeneous expressions of T-antigen in immortal clones from SV40-infected human cells are consistent with our observation [13, 35, 51, 63, 86] (see also Background).

We conclude from the non-correlations of T-antigen with the immortal neoplastic clones studied by us here and previously by others (Background), that T-antigen is not necessary to maintain neoplastic transformation and immortality. If the T-antigen were necessary for neoplastic transformation and immortality, then it should be present in all cells of a clone, just like the individual clonogenic karyotypes described above. Moreover, its level should have been equalized for optimal neoplastic concentration in all cells of the immortal F1 and F4 lines after selections for autonomy over the 200 generations since their isolation (see above). 

The over 20 previous studies listed in the Background, which reported SV40-induced neoplastic human, hamster, mouse and rat clones without viral genes and T-antigens lend further definitive support to this conclusion.

In the following we have tested the generality of the theory that SV40 transforms cells indirectly by inducing aneuploidy, which catalyzes spontaneous karyotypic evolutions of rare immortal neoplastic clones in rat and mouse cells.

### II. Transformation of rat cells by SV40

Next we tested, whether the karyotypic mechanism of transformation described for human mesothelial cells also applies to the formation of neoplastic clones from SV40-infected rat cells. For this purpose we studied primary rat lung cells infected with SV40 virus at a multiplicity of two, as described above for human cells and previously by others for primary rat cells [3, 41, 88].

### Karyotypes of SV40-infected preneoplastic rat lung cells

To test SV40-infected lung cells for the preneoplastic aneuploidy predicted by the karyotypic theory, the karyotypes of infected cells were determined three weeks after infection. This time point was chosen, because confluent cultures of rat lung cells infected at a multiplicity two and passaged twice at 3-fold dilutions formed foci three weeks after infection (see next paragraph). At that time we analyzed the karyotypes of the non-transformed, inter-focal regions of the infected culture for virus-induced preneoplastic aneuploidy. As can be seen in Table 3, 65 % of the infected, non-transformed rat cells contained randomly aneuploid karyotypes at that time, by contrast un-infected controls were 95 % diploid (not shown). This result extended the findings of high percentages of preneoplastic aneuploidies in SV40-infected human mesothelial cells, described above in Tables 1 and 2 and in the Background. We conclude that SV40 induces preneoplastic aneuploidy in rat cells, just as it did in the human cells described above.

**Table 3:** Karyotypes of rat lung cells 3 weeks after infection with SV40: 13 aneuploid per 20

Karyotypes	1	2	3	4	5	6	7	8	9	10	11	12	13
Total no of chromosomes	45	42	43	43	43	44	42	43	43	44	42	70	86
Chromosomes													
1	2	2	2	2	2	2	2	2	2	2	2	3	4
2	2	2	2	2	2	2	1	2	2	2	2	4	4
3	2	3	2	2	3	2	3	3	2	2	2	3	6
4	2	2	2	2	2	2	2	2	2	2	2	2	4
5	2	2	2	2	2	2	2	2	2	2	1	4	4
6	2	2	2	2	2	2	2	2	2	2	2	4	4
7	2	2	2	2	2	2	2	2	2	2	2	4	4
8	2	2	2	2	2	2	2	2	2	2	2	4	4
9	2	2	2	2	2	2	2	2	2	2	2	4	4
10	1	2	2	2	2	2	2	2	2	2	2	4	4
11	3	2	2	2	2	2	2	2	2	2	2	2	4
12	1	2	2	2	2	2	2	2	2	2	2	3	4
13	2	2	2	2	2	2	2	2	2	2	2	3	4
14	2	2	2	2	2	2	2	2	2	2	2	2	4
15	2	2	2	2	2	2	2	2	2	2	2	3	4
16	2	1	2	2	2	2	2	2	2	2	2	3	4
17	2	2	2	2	2	2	2	2	2	2	2	2	4
18	2	2	2	2	2	2	2	2	2	2	2	4	4
19	2	2	2	2	2	2	2	2	2	2	2	4	4
20	2	2	2	2	2	2	1	2	2	2	2	4	4
X	2	2	2	2	2	2	1	2	2	2	2	4	4
Y	0	0	0	0	0	0	0	0	0	0	0	0	0
der(3;12)	0	0	0	0	0	1	0	0	1	0	0	0	0
del(2)(q?)	0	0	0	0	0	0	1	0	0	0	0	0	0
dic(X;20)	0	0	0	0	0	0	1	0	0	0	0	0	0
der(5)	0	0	0	0	0	0	0	0	0	0	1	0	0
der(12;10)	1	0	0	0	0	0	0	0	0	0	0	0	0
der(10)	1	0	0	0	0	0	0	0	0	0	0	0	0
dic(11;17)	1	0	0	0	0	0	0	0	0	0	0	0	0
del(17q)	1	0	0	0	0	0	0	0	0	0	0	0	0
mar(?)	0	0	1	1	0	1	0	0	0	0	0	0	0
der(3;2)	0	0	0	0	0	0	0	0	0	1	0	0	0
del(14q)	0	0	0	0	0	0	0	0	0	1	0	0	0

### Phenotypes and karyotypes of focal colonies of transformed rat cells

**Fig. 9:**
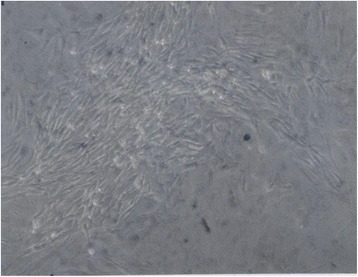
Focus of transformed cells from a culture of rat lung cells three weeks after infection with SV40. This focus was one of 108 that arose in confluent secondary cultures of rat lung cells three weeks after infection of a primary culture with SV40 virus. The micrograph was taken at 120X magnification. Details of preparing the culture are described in the text

Three weeks after infection of 10^6 rat lung cells and their subsequent expansion to confluent cultures, as described above, a total of 108 foci of morphologically transformed cells arose from three confluent 10-cm dishes of infected rat cells. This time course from infection to focus formation in rat cells confirms and extends prior studies [39, 83]. A typical focus arising from the confluent background of the virus-infected rat cells is shown in Fig. 9. The yield of 108 focal colonies of transformed cells corresponds to clonogenic transformation of only one in 10,000 of the 10^6 originally infected cells. This low probability of neoplastic transformation of SV40-infected rat cells is also consistent with that observed in prior studies of neoplastic transformation of rat [41, 88] and of human mesothelial cells described by others including Bochetta et al., who prepared the F1 an F4 lines studied above [21], (see also Background).

#### Morphologically distinct phenotypes of neoplastic rat clones

**Fig. 10:**
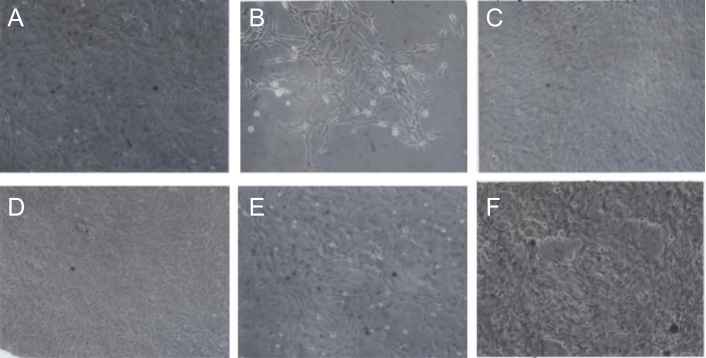
Individual cell morphologies of six focal colonies from cultured primary rat lung cells, three weeks after infection with SV40. To test the theory that the karyotypes of individual neoplastic clones encode individual phenotypes, we analyzed the cellular morphologies of six focal colonies from SV40-infected rat lung cells (see example in Fig. 9). As can be seen by the 120X magnification of cultures of the six focal colonies, F8 (**a**), F33 (**b**), F3 (**c**), F100 (**d**), F10 (**e**) and FC1 (**f**), all clones had individual cell morphologies. This result indicates that individual karyotypes, rather than common viral genes, encode the individual phenotypes of the distinct neoplastic clones

To determine, whether the focal colonies of SV40-infected rat cells contain the predicted individual clonal phenotypes, cultures of six colonies termed, F8, F33, F3, F100, F10 and FC1 were investigated. Micrographs at 120X magnification of these six cultures show in Fig. 10 that each colony had its own, rather uniform and thus quasi-clonal cell morphology. This result also confirmed previous descriptions of clonal individualities [41, 88]. Moreover, the estimated growth rates of individual colonies were clone-specific like those described previously by others [3, 17, 41, 88]. The growth rate of the F100 clone was stable over 50 passages corresponding to over 100 cell generations, consistent with neoplastic immortality. In sum, these results confirmed the prediction of the karyotypic theory that individual neoplastic clones have individual clonal phenotypes (Fig. 1).

#### Individual karyotypes of neoplastic rat clones

**Fig. 11:**
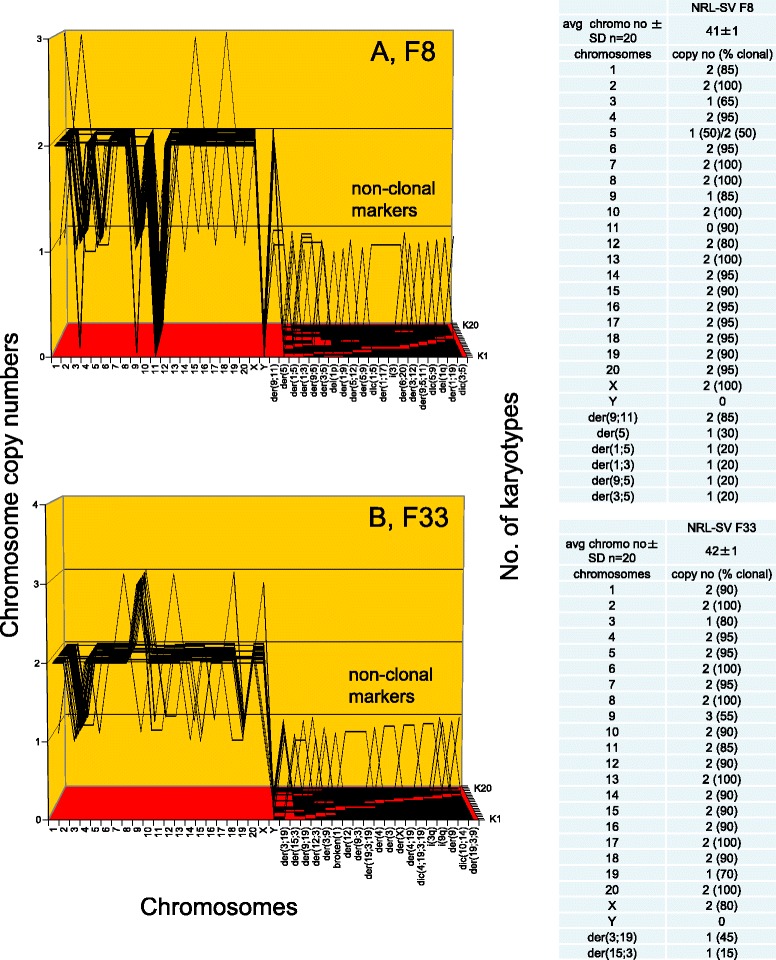
Karyotype arrays of six morphologically distinct focal colonies derived from SV40-infected rat lung cells: first pair of three. To test the theory that individual clonal karyotypes encode the individual phenotypes of neoplastic clones, the karyotype arrays the six morphologically distinct focal rat colonies F8, F33, F3, F100 and FC1, shown above in Fig. 10, were compared to each other in three separate Figures, namely 11, 12 and 13. **a** shows that F8 has a near-diploid karyotype with 41 chromosomes that were 85–100 % clonal. Accordingly, the F8-chromosomes formed a quasi-clonal F8-specific karyotype array. The non-clonal fraction of chromosomes included several partially clonal and several non-clonal marker chromosomes, indicative of ongoing karyotypic variation (see Fig. 1). **b** shows that F33 contained a pseudo-diploid karyotype with 42 chromosomes that were 70–100 % clonal. Accordingly, the F33 chromosomes also formed an individual quasi-clonal array that was different from that of F8. The individualities of these two karyotype arrays thus support the theory that individual karyotypes encode the individual phenotypes of neoplastic clones. (The karyotype arrays of the remaining four rat colonies are shown in Figs. 12 and 13)

To test for the predicted clonal karyotypic origins of the six rat colonies F8, F33, F3, F100, F10 and FC1 shown in Fig. 10, we used the karyotype array technique that was introduced for this purpose above in Fig. 5 and Section I. The karyotype arrays of these six neoplastic rat colonies are shown below in three figures, each depicting the arrays of two of the six rat colonies with the following results:

Figure 11a and the attached table shows that F8 contains a near-diploid karyotype with 41 chromosomes, which were 85–100 % clonal. (The normal Norwegian rat contains 42 chromosomes.) Accordingly the F8-chromosomes formed a quasi-clonal F8-specific karyotype array. In addition F8 contained several partially clonal and several non-clonal marker chromosomes, indicative of ongoing karyotypic variation (explained in Fig. 1). Fig. 11b shows that F33 contains a pseudo-diploid karyotype with 42 chromosomes that were 70–100 % clonal. Accordingly the F33 chromosomes also formed an individual quasi-clonal karyotype array, which was different from that of F8. The distinct individualities of these two karyotype arrays thus support the theory that neoplastic clones have individual karyotypes that encode individual phenotypes.

**Fig. 12:**
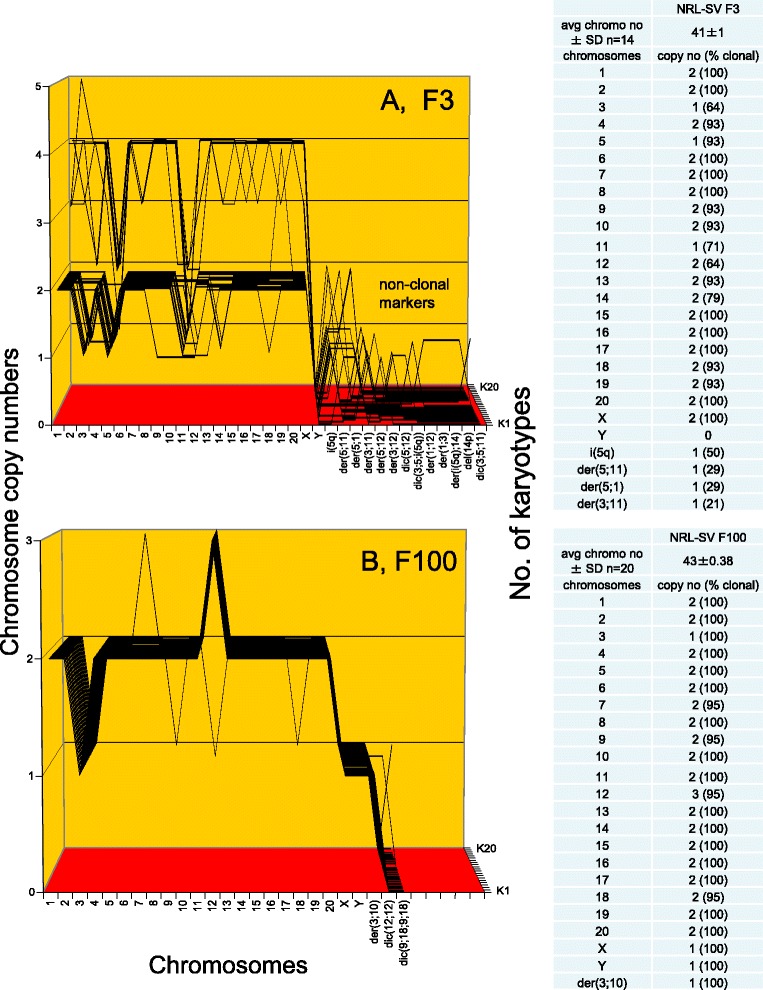
Karyotype arrays of six morphologically distinct focal colonies from SV40-infected rat lung cells: second pair of three. To test the theory that individual clonal karyotypes encode the individual phenotypes, two more of the six morphologically distinct rat clones described in Fig. 10 were compared. **a** shows that F3 has a near-diploid karyotype of 41 chromosomes that were 70–100 % clonal. Accordingly, the F3 chromosomes formed an individual, quasi-clonal karyotype array, which was different from those of F8 and F33 shown in Fig. 11. A third of the F3 cells had near-tetraploid karyotypes that appeared to be duplications of the predominant near-diploid F3 karyotypes. **b** shows that F100 has a near-diploid karyotype with 43 chromosomes that were 95–100 % clonal. Accordingly the F100 chromosomes also formed a quasi-clonal F100 karyotype array, which was different from those of F3, F8 and F33. Thus the karyotypes of four distinct neoplastic rat clones support the theory that individual karyotypes encode the individual phenotypes of neoplastic clones

Figure 12a shows that F3 contains a near-diploid karyotype of 41 chromosomes that were 70-100 % clonal. Accordingly, the F3-chromosomes formed an individual quasi-clonal F3-specific karyotype array, which was different from those of F8 and F33 shown in Fig. 11. A third of the F3 cells had near-tetraploid karyotypes that appeared to be duplications of the predominant near-diploid F3 karyotypes. Figure 12b shows that F100 also contains a near-diploid karyotype with 43 chromosomes that were 95-100 % clonal. The very high clonality of the F100 colony may be a result of stabilizing selections during its long passage history. F100 dates from an early pilot experiment conducted before the other rat clones were prepared. Accordingly the F100-chromosomes also formed an individual clonal F100-specific karyotype array, which was different from those of F3, F8 and F33. Thus, the karyotypes of four distinct neoplastic rat clones support the theory that individual karyotypes encode the individual phenotypes of neoplastic clones.

**Fig. 13:**
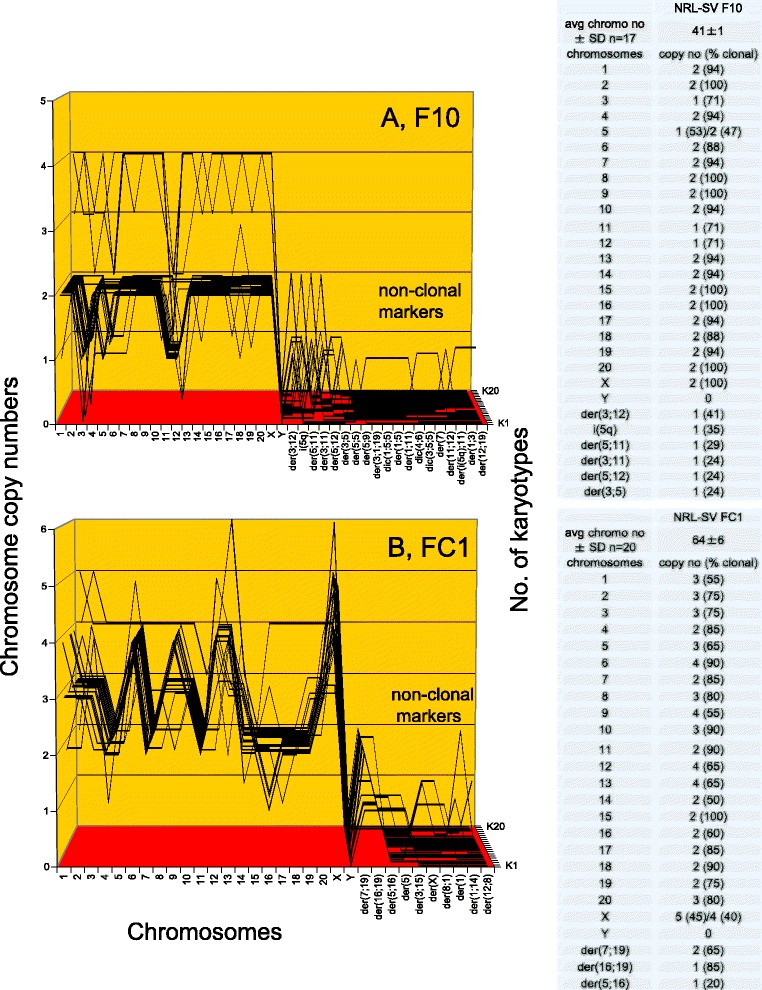
Karyotype arrays of six morphologically distinct focal colonies derived from SV40-infected rat lung cells: third pair of three. To test the theory that individual clonal karyotypes encode individual phenotypes, we analyzed and compared the arrays of a third pair of six individually distinct rat focal colonies. **a** shows that F10 has a near-diploid karyotype with an average number of 41 chromosomes that were between 70 and 100 % clonal. Accordingly they formed a quasi-clonal F10-specific array, which is different from those of the F8, F33, F3 and F100 colonies. The F10 clone also includes a minor (15 %) tetraploid variant, similar to that found in clone F3 described above (Fig. 12a). **b** shows that clone FC1 has a near triploid karyotype with 64 chromosomes that were between 55 and 100 % clonal. Accordingly they formed a quasi-clonal FC1-specific array, which differs from those of all five sister colonies described above and in Figs. 11 and 12. Thus the individual karyotypes of the six phenotypically distinct rat clones support the theory that individual karyotypes encode the individual phenotypes of neoplastic clones

Figure 13a shows that F10 contains a near-diploid karyotype with an average number of 41 chromosomes that were between 70 and 100 % clonal. Accordingly they formed a quasi-clonal array, which was different from those of colonies F8, F33, F3 and F100. The F10 clone also included a minor (15 %) tetraploid variant, similar to that found in clone F3 described above (Fig. 12a). Fig. 13b shows that clone FC1 contains a near triploid karyotype with 64 chromosomes that were between 55 and 100 % clonal. Accordingly the FC1 chromosomes formed an individual quasi-clonal array, which differed from those of all five sister colonies described above and in Figs. 11 and 12.

In sum, the individual karyotype arrays of all six morphologically distinct rat clones support the theory that individual clonal karyotypes generate and encode the individual phenotypes of neoplastic clones.

### Non-correlations between SV40 T-antigen and the cells of the neoplastic rat clones F3 and F100

**Fig. 14:**
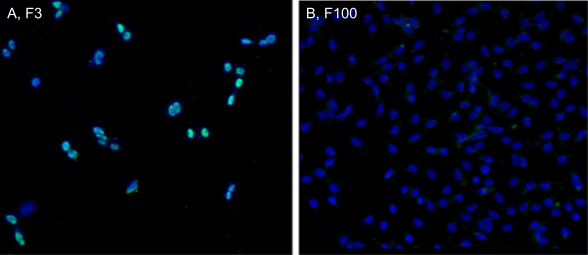
Non-correlations between the cells of two SV40-induced neoplastic clones of rat lung cells and SV40 T-antigen. To test the prediction of the karyotypic theory that the clonal karyotypes of SV40-induced neoplastic clones are sufficient to generate and maintain neoplastic clones, independent of the viral T-antigen, we analyzed the cells of the SV40-induced neoplastic rat clones F3 and F100 (described in Figs. 10 and 12) for the presence of viral T-antigen with green-labeled antibodies, as described above for Fig. 8. **a** shows that about 30 % of the F3 cells were T-antigen negative, while the remaining 70 % were heterogeneous, expressing T-antigen between very low to relatively high levels. **b** shows that all cells of the neoplastic rat clone F100 were T-antigen negative under the conditions of our test (Methods). We concluded from the absence of detectable T-antigen in F100 and the absence or heterogeneous presence of T-antigen in F3 cells that T-antigen is not necessary to maintain neoplastic transformation of SV40-induced neoplastic rat clones, as predicted by the karyotypic theory

It may be argued as above (Fig. 8) that the individual clonal karyotypes of virus-induced neoplastic rat clones may depend on SV40 genes for neoplastic transformation. In view of this we tested the neoplastic clones F3 and F100 for the presence of T-antigen, as described above for Fig. 8. As shown in Fig. 14a, we found that SV40 T-antigen is heterogeneously distributed among the cells of the F3 clone: some cells were T-antigen-free and others contained various low to high levels of T-antigen. By contrast, we found no T-antigen in F100 cells under our conditions, as shown in Fig. 14b. The absence of the karyotype-destabilizing effect of the T-antigen may also explain the high clonality of F100 (see Fig. 12b).

Because of these non-correlations between T-antigen and neoplastic rat cells prepared here and those observed above with neoplastic human cells (Fig. 8), we conclude that T-antigen is not necessary for the neoplastic proliferation of SV40-induced immortal human and rat clones (Section I, Fig. 8). This conclusion is supported by dozens of other studies describing SV40-induced neoplastic clones of human or rodent cells that either contain fractions of T-antigen-negative cells or are entirely T-antigen-free (Background).

### In sum karyotypic analysis of neoplastic transformation of rat cells by SV40 virus confirms karyotypic theory of viral transformation

The karyotypic clonality and individuality of six focal rat colonies thus confirms once more the prediction of the karyotypic theory that individual karyotypes are the genetic origins of these neoplastic clones and maintain the individualities of these clones via complex transcriptomes [37, 38, 43, 44]`– independent of SV40 genes. Since viral genes are not cancer-specific, not likely to generate individuality, not present in all neoplastic cells and are too few to explain the endless individualities of virus-induced neoplastic clones and tumors described here and previously (Background), we conclude that the individual clonal karyotypes of SV40-induced neoplastic clones are sufficient for neoplastic transformation.

In the following we have tested the karyotypic theory of viral transformation in SV40-infected mouse embryo cells.

### III. Transformation of mouse embryo cells by SV40

Last we test whether the karyotypic mechanism of transformation described for human mesothelial and rat lung cells also applies to the formation of neoplastic clones from SV40-infected mouse embryo cells. For this purpose we studied mouse embryo cells infected with SV40 virus, as described above for rat cells and previously for mouse lung and tail cells [89].

### Karyotypes of SV40-infected preneoplastic mouse embryo cells

To determine, whether SV40 induces preneoplastic aneuploidy in mouse cells as predicted by the karyotypic theory, we examined cultures derived from 10^6 mouse embryo cells infected with SV40 at a multiplicity of two as described above for the infection of rat cells. About three weeks after infection, when rare foci of transformed cells first appeared (see next paragraph), we karyotyped the non-transformed, inter-focal regions of the SV40-infected mouse embryo cultures to test for the predicted preneoplastic aneuploidy.

As shown in Table 4, 65 % or 13 of 20 cells of the infected, non-transformed mouse cells contained randomly aneuploid karyotypes at that time, compared to less than 5 % in uninfected controls (not shown). This high level of aneuploidization of mouse cells by SV40 is very similar to those described above for SV40-infected human and rat cells at this stage of infection (Tables 1, 2 and 3; See also Background). At variance with human and rat cells, 77 % or 10 of the 13 karyotypes of aneuploid mouse cells were near tetraploid.

**Table 4:** Karyotypes of mouse embryonic cells 3 weeks after infection with SV40: 13 aneuploid per 20

Karyotypes	1	2	3	4	5	6	7	8	9	10	11	12	13
Total no of chromosomes	36	40	39	74	74	81	79	78	79	79	78	79	80
Chromosomes													
1	2	1	1	4	3	4	4	4	4	4	4	4	4
2	2	2	2	4	4	4	4	4	4	4	4	4	4
3	2	2	2	3	3	4	4	4	4	4	3	4	4
4	2	2	2	3	3	4	4	4	4	3	4	4	4
5	2	2	1	3	4	4	4	4	4	4	4	4	4
6	1	2	2	4	3	4	4	2	4	4	4	4	4
7	2	2	2	4	4	4	4	4	4	4	4	4	4
8	2	2	2	4	4	4	4	4	4	4	4	4	4
9	1	1	1	3	3	4	4	4	4	4	4	4	4
10	2	1	1	4	3	4	4	4	4	4	4	4	4
11	2	2	1	3	4	4	4	4	4	4	4	4	4
12	1	2	2	4	4	4	4	4	4	4	4	3	4
13	2	2	2	4	4	4	4	4	4	4	4	5	4
14	1	2	2	4	4	4	4	4	4	4	3	4	4
15	2	2	2	4	4	4	4	4	4	4	4	4	4
16	2	2	2	3	4	4	4	4	4	4	4	4	4
17	1	2	2	4	4	5	4	4	4	4	4	3	4
18	2	2	2	4	4	4	3	4	4	4	4	4	4
19	2	2	2	4	3	4	4	4	4	4	4	4	4
X	1	1	1	2	2	2	2	2	2	2	2	2	2
Y	1	1	1	2	2	2	2	2	1	2	2	2	2
dup(Xq)	0	0	0	0	1	0	0	0	0	0	0	0	0
del(9)(q)	1	0	0	0	0	0	0	0	0	0	0	0	0
der(1;9)	0	1	1	0	0	0	0	0	0	0	0	0	0
der(10;11)	0	1	0	0	0	0	0	0	0	0	0	0	0
der(10)	0	1	1	0	0	0	0	0	0	0	0	0	0
der(5)	0	0	1	0	0	0	0	0	0	0	0	0	0
der(11)	0	0	1	0	0	0	0	0	0	0	0	0	0

### Phenotypes and karyotypes of focal colonies of transformed mouse cells

Between three and four weeks after infection of the primary culture of 10^6 mouse embryo cells about 50 distinct foci of morphologically transformed cells appeared in confluent subcultures, which were expanded and maintained as described above for the rat cultures. This yield of 50 foci corresponded to clonogenic neoplastic transformation of about one per 20,000 of the one million originally infected cells. This low yield of clonogenic transformation is in close agreement with the yields of about one or less per 10,000 SV40-infected rat and human mesothelial cells described above and in Background.

#### Phenotypes of neoplastic mouse clones

**Fig. 15:**
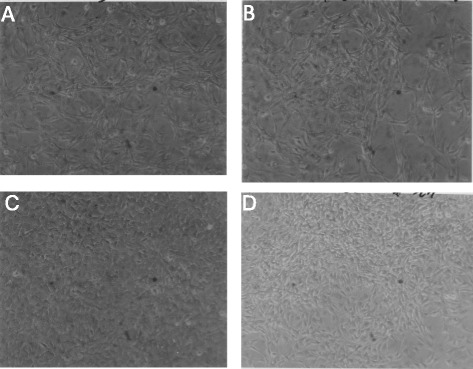
Distinct cell morphologies of four Individual focal colonies from SV40-infected mouse embryo cells. To test the theory that individual karyotypes encode neoplastic clones with individual phenotypes, we analyzed the cellular morphologies of four focal colonies that arose from SV40-infected mouse embryo cells. As seen by 125-fold magnification of cultures of four such colonies, F1 (**a**), F9 (**b**), F10 (**c**) and F11 (**d**), each clone had an individual and apparently clonal cell morphology. This result indicates that individual genotypes, rather than common SV40 genes encode the morphologies of these virus-induced colonies

We selected four of these foci, F1, F9, F10 and F11 to determine whether they contain the individual clonal phenotypes and karyotypes predicted by the karyotypic theory. As can be seen in Fig. 15, all four focal colonies from SV40-infected mouse cells had individual and clonal cell morphologies, as was the case for the rat colonies described above in Fig. 10. These focal mouse colonies also grew at individual rates. For example, the growth rate of F9 was relatively low compared to those of F10 and F11. Moreover, preliminary tests of the predicted immortality of F10 (Fig. 1) showed that this clone survived over 40 cell generations in culture without any loss of viability. In fact the growth rate of F10 seemed to increase during these passages in culture confirming reports that transformation to autonomous growth and immortality is easier to achieve with SV40-infected mouse cells than with human cells [13, 17, 108].

#### Karyotypes of neoplastic mouse clones

To test for the predicted clonal karyotypic origins of the four transformed colonies F1, F9, F10 and F11 of SV40-infected mouse embryo cells shown in Fig. 15, we prepared their karyotype arrays as described above for Figs. 5, 6, 7, 11, 12 and 13. The results are shown in the following two Figures, each depicting the arrays of two of the four focal colonies.

**Fig. 16:**
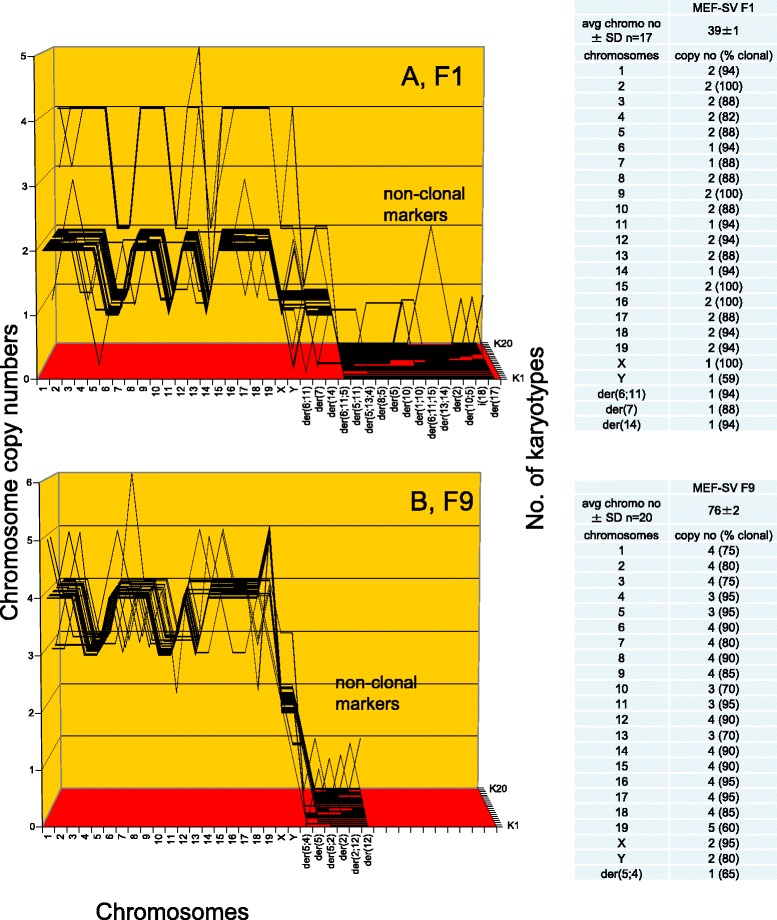
Karyotype arrays of four morphologically distinct neoplastic colonies derived from SV40-infected mouse embryo cells: first two of four. To test the theory that individual clonal karyotypes encode the phenotypes of individual neoplastic clones from SV40-infected mouse cells, the karyotype arrays of the four focal mouse colonies F1, F9, F10 and F11, shown in Fig. 15 were compared to each other in two separate figures, namely 16 and 17. **a** shows that F1 has a near diploid karyotype with 39 chromosomes including three marker chromosomes, which were 88–100 % clonal. Accordingly the F1 chromosomes formed a quasi-clonal F1-specific karyotype array. **b** shows that F9 has a hypo-tetraploid karyotype with 76 chromosomes, which 60–95 % clonal. Accordingly the F9 chromosomes formed a quasi-clonal F9-specific karyotype array, which is different from that of F1. The 1-to-1 karyotype-phenotype correlation of F1 and F9 thus supports the theory that individual karyotypes, rather than common viral genes, encode the individual phenotypes of the SV40-induced neoplastic mouse clones

Figure 16a and the attached table shows that the F1 mouse colony has a near diploid karyotype with an average number of 39 chromosomes, which were 82 to 100 % clonal. (The normal mouse contains 40 chromosomes.) Accordingly, the 39 F1-chromosomes formed a quasi-clonal F1-specific karyotype array, which is shown in Fig. 16a. The F1 colony also included a minor (15 %) tetraploid variant, much like the two rat clones described above in Figs. 11a and 12a. Figure 16b shows that the F9 colony has a hypo-tetraploid karyotype with an average number of 76 chromosomes, which were 65 to 95 % clonal. Accordingly the F9-chromosomes also formed an individual quasi-clonal F9-specific array, which was different from that of F1.

**Fig. 17:**
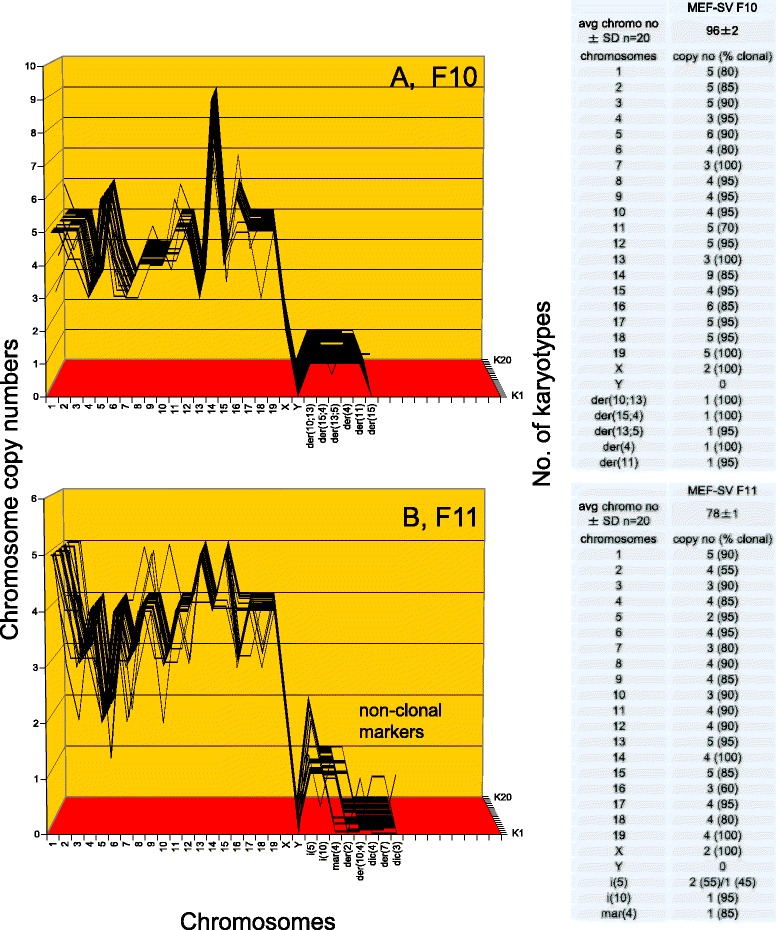
Karyotype arrays of four morphologically distinct neoplastic colonies derived from SV40-infected mouse embryo cells: second pair of two. To test the theory that individual clonal karyotypes encode individual phenotypes, the karyotypes of two of four mouse colonies, namely F10 and F11 were compared here. **a** shows that F10 has a hyper-tetraploid karyotype with 96 chromosomes that were 70–100 % clonal. Accordingly, the F10 chromosomes formed an F10-specific quasi-clonal array, which is different from those of F1 and F9. **b** shows that F11 has a near tetraploid karyotype with an average number of 78 chromosomes, which were 55–100 % clonal. Accordingly the F11 chromosomes also formed an individual quasi-clonal array, which is different from those of all three sister colonies, F1, F9, and F10 tested above. The individual karyotypes of the four phenotypically distinct mouse clones thus support the theory that individual karyotypes encode the individual phenotypes of neoplastic clones

Figure 17a and the attached table shows that the F10 mouse colony has a hyper-tetraploid karyotype with an average number of 96 chromosomes, which were 70 to 100 % clonal. Accordingly the F10-chromosomes formed an individual quasi-clonal F10-specific array, which differed from those of F1 and F9. Figure 16b shows that F11 has a near-tetraploid karyotype with an average of 78 chromosomes, which were 85 to 100 % clonal with the exception of chromosome 4 that was only 55 % clonal. Accordingly, the F11-chromosomes also formed an individual, quasi-clonal F11-specific array, which differed from those of all three sister clones.

### In sum karyotypic analysis of neoplastic transformation of mouse cells by SV40 virus confirms the karyotypic theory of viral transformation

Thus all four morphologically distinct focal colonies of SV40-infected mouse cells analyzed formed highly clonal, individual karyotype arrays – similar to the individual neoplastic clones of SV40-infected human and rat cells described above. Moreover, there is also evidence in the literature that virus-induced neoplastic mouse clones each have highly complex, individual transcriptomes [37, 38], which directly confirms the view that individual karyotypes encode the individual phenotypes of neoplastic clones via individual transcriptomes. The clonality and individuality of the karyotypes of four focal mouse colonies thus confirms once more the prediction of the karyotypic theory that individual karyotypes are the genetic origins of these neoplastic clones, and maintain the individualities of these clones – rather than common viral T-antigens, which are present in all pre-neoplastic cells and which are unlikely to encode the complex individual transcriptomes and phenotypes of neoplastic clones.

Moreover, the facts that (1) three of the four neoplastic mouse clones were near tetraploid and a fourth was partially tetraploid, and that (2) the majority of preneoplastic karyotypes of SV40-infected mouse cells was also tetraploid (Table 4) support the prediction of our theory that cells with preneoplastic aneuploidies are the precursors of neoplastic clones (Fig. 1).

## Conclusions

This study was undertaken to test the theory that SV40 induces cancer indirectly by inducing preneoplastic aneuploidy, much like conventional carcinogens [97, 101, 109, 110]. Since aneuploidy unbalances thousands of genes, it destabilizes the karyotype and thus catalyzes automatic evolutions of new karyotypes and transcriptomes including rare cancer-specific karyotypes and transcriptomes at very low rates [97]. This theory is outlined in the Background and graphically summarized in Fig. 1. The theory predicts that despite their destabilizing congenital aneuploidies cancer karyotypes are stabilized or immortalized within narrow karyotype-specific margins of variations by selections for cancer-specific autonomy of growth [97, 101, 102], (Fig. 1). Moreover, the theory predicts that the abnormal karyotypes of cancers generate highly complex, individual transcriptomes with hundreds of abnormally expressed mRNAs and thus highly complex new phenotypes. This prediction has already been confirmed in several studies of SV40-induced neoplastic clones of human and mouse cells [37, 38, 43, 44].

To test this karyotypic virus-cancer theory, we have analyzed the karyotypes and phenotypes of SV40-infected human, rat, and mouse cells from infection to the evolution of rare immortal neoplastic clones. In all three systems we found the predicted (1) preneoplastic aneuploidies, (2) neoplastic transformation at low rates, namely less than one of 10,000 infected cells, which is incompatible with direct viral transformation, (3) neoplastic clones with individual clonal karyotypes, which define the genetic origin [101, 102] and maintain the complex individual phenotypes of neoplastic clones via complex individual transcriptomes [37, 38, 43, 44], (4) variations of cancer phenotypes, such as cell morphology and drug-resistance, that correlated 1-to-1 with variations of karyotypes, and (5) immortality of virus-induced neoplastic clones surviving over 200 generations, which is cancer-specific [89] and is analogous to the immortality of non-viral cancers [89, 97] – and indeed to the immortality of all normal species [92–95]. But, we found no consistent correlations between neoplastic cells and SV40 T-antigen.

We also tested our theory for its ability to explain as yet unclear roles of viruses in two other cancer systems:

First, our theory confirmed the “assumption” of Rous and Beard studying Shope rabbit fibroma virus in 1935, “The virus is the immediate cause for carcinosis; yet compatible with the assumption that it merely provides an essential, preliminary cell disturbance.” [111]. Not surprisingly, in view of the karyotypic theory, Palmer [112] and subsequently McMichael, Wagner, Nowell and Hungerford [113] found in 1959 and 1963 that the carcinomas induced by the Shope-fibroma virus have individual karyotypes. But without a theory for the karyotypic individuality of cancers McMichael et al. thought “their significance with respect to the subsequent development of malignancy remains obscure” [113].

Second, the karyotypic theory predicts a testable target for the poorly defined “hit-and-run” hypothesis of human Adenovirus-induced but Adenovirus-free experimental tumors and neoplastic clones [114, 115] – namely clonal cancer-specific karyotypes like those identified here in SV40-induced but SV40-free and T-antigen-free neoplastic clones and tumors. In addition our theory provides a plausible explanation for the induction of preneoplastic aneuploidy also induced by Adenoviruses in human cells [116, 117].

In view of our experimental and theoretical tests of the karyotypic theory, we conclude that SV40 and likely Shope-fibroma and Adeno-viruses induce cancers indirectly, by inducing preneoplastic aneuploidy, which catalyzes spontaneous evolution of virus-independent cancer karyotypes at low rates – much like conventional carcinogens induce carcinogen-independent cancers by inducing preneoplastic aneuploidy [101].

## Methods

### Infection and transformation of primary human mesothelial, rat lung and mouse embryo cells with SV40 tumor virus

Subconfluent cultures of primary human mesothelial cells [21], rat lung cells (prepared from a 0.5 to 1-year old Sprague Dawley rat from the Office of Laboratory Animal Care (University of California at Berkeley) and primary mouse embryo cells from the Tissue Culture Facility at UC Berkeley (Barker Hall, UCB) were grown in RPMI 1640 medium (Sigma Co.) supplemented with 3 to 5 % fetal calf serum and antibiotics as described previously [101, 102]. Subconfluent cultures of about 500,000 cells per 5 cm-culture dishes in 3 ml medium were infected at multiplicities of 10 for human cells and at multiplicities of 2 for rat and mouse cells as described previously [25].

### Karyotype analysis

One to two days before karyotyping, cells were seeded at about 50 % confluence in a 5-cm culture dish with 3 ml medium containing 3 to 5 % fetal calf serum. After reaching ~75 % confluence, 250 ng colcemid in 25 μl solution (KaryoMax, Gibco) was added to 3 ml medium. The culture was then incubated at 37 °C for 4–8 h. Subsequently cells were dissociated with trypsin, washed once in 3 ml of physiological saline and then incubated in 0.075 molar KCl at 37 °C for 15 min. The cell suspension was then cooled in ice-water, mixed (‘prefixed’) with 0.1 volume of the freshly mixed glacial acetic acid-methanol (1:3, vol. per vol.) and centrifuged at 800 g for 6 min at room temperature. The cell pellet was then suspended in about 100 μl supernatant and mixed drop-wise with 5 ml of the ice-cold acetic acid-methanol solution and incubated at room temperature for 15 min or overnight at -20C. This cell suspension was then pelleted once more as above and re-suspended in a small volume of the acetic acid-methanol solution. An aliquot of a visually turbid suspension was then transferred with a micropipette tip to a glass microscope slide, allowed to evaporate at room and inspected under the microscope at x200 for a an adequate non-overlapping density of metaphase chromosomes. Metaphase chromosomes attached to glass slides were then hybridized to color-coded, chromosome-specific DNA probes as described by the manufacturer (MetaSystems, Newton, MA 02458). Karyotypes were analyzed under a fluorescence microscope as described by us previously [17, 49].

### T-antigen staining

Anti-SV40 T antigen antibody and Alexa Fluor 488 goat anti-mouse IgG were purchased from Abcam (Boston, MA). The SV40-transformed human and rat cell lines that we tested were grown on microscope slides or cell culture dishes, washed with phosphate-buffered saline, and wet cultures were fixed with freshly prepared, cold methanol-acetic acid (3:1) for 15 minutes. The cultures were then rinsed again with saline and reacted with antibodies according to the manufacturer’s protocol.

All work on human and animal cell cultures has been approved by the Environmental Health and Safety Committee of the University of California at Berkeley and by the Lawrence Berkeley Laboratory at Berkeley.

### Note added in proof 

In an effort to confirm our evidence for T-antigen-negative cells of the immortal SV40-induced human mesothelial F1 line, we analyzed 25 single-cell-derived colonies of the puromycin-resistant variant of the F1-line described in Figure 6. We found that among 25 such colonies, 5 were T-antigen positive, 7 were positive but with different degrees of positivity and 13 were entirely negative. This result confirms that, based on our test, T-antigen is not necessary for neoplastic transformation and for immortality of F1. Moreover, we note that the karyotypic cancer theory explains the high individual multiplicities of over- and under-expressed cellular proteins of SV40-transformed neoplastic cells [118].
